# National survey on current situation of critical value reporting in 973 laboratories in China

**DOI:** 10.11613/BM.2017.030707

**Published:** 2017-10-15

**Authors:** Yang Fei, Haijian Zhao, Wei Wang, Falin He, Kun Zhong, Shuai Yuan, Zhiguo Wang

**Affiliations:** National Center for Clinical Laboratories/Beijing Engineering Research Medicine, Beijing Hospital, National Center of Gerontology, Beijing, P.R. China

**Keywords:** critical value, quality indicators, extra-analytical phase

## Abstract

**Introduction:**

The aim of the study was to investigate the state-of-the-art of the performance of critical value reporting and provide recommendations for laboratories setting critical value reporting time frames.

**Materials and methods:**

The National Centre for Clinical Laboratories in China initiated a critical value reporting investigation in 2015. A questionnaire related to critical value reporting policy was sent to 1589 clinical laboratories in China online. The questionnaire consisted of a set of questions related to critical value reporting policy and a set of questions related to timeliness of critical value reporting. The survey data were collected between March and April 2015.

**Results:**

A total survey response rate was 61.2%. The critical value unreported rate, unreported timely rate, and clinical unacknowledged rate of more than half of participants were all 0.0%. More than 75.0% of participants could report half of critical values to clinicians within 20 minutes and could report 90.0% of critical values to clinicians within 25 minutes (from result validation to result communication to the clinician). The median of target critical value reporting time was 15 minutes. “Reporting omission caused by laboratory staff”, “communications equipment failure to connect”, and “uncompleted application form without contact information of clinician” were the three major reasons for unreported critical value.

**Conclusions:**

The majority of laboratories can report critical values to responsible clinical staff within 25 minutes. Thus, this value could be recommended as suitable critical value reporting time frame for biochemistry laboratories in China. However, careful monitoring of the complete reporting process and improvement of information systems should ensure further improvement of critical value reporting timeliness.

## Introduction

The term “critical result” was first proposed by Dr. George D. Lundberg over 40 years ago. He suggested that patients with critical test results were in a life-threatening situation if action was not taken quickly, thus critical results should be communicated to responsible caregivers without delay (*1*). As timely release and reporting of critical results is essential for optimal clinical care, critical results’ reporting has drawn great attention. It has not only become a laboratory accreditation requirement, but also a quality practice to improve service quality in the clinical laboratory (*2*, *3*).

The International Standardization Organization (ISO) 15189 specified the requirement on timeliness of critical values reporting (*2*). The National Patients Safety Goals in China, Hospital Management Evaluation Guideline, and the Evaluation Standards for General Hospitals also addressed the importance of timeliness reporting of critical values (*4*). Although timeliness of critical value reporting has been specified, none of them described the time frames of critical value reporting in detail. The Clinical and Laboratory Standards Institute (CLSI) document GP47 recommended that “immediately life-threatening, critical-risk results should be reported within one hour of identification or availability of the result” (*5*). It is obviously inappropriate to use recommendation of one hour without verification. Besides, quality specifications can be set based on the “state-of-the-art” (*6*). Based on a College of American Pathologists (CAP) Q-Probes study, a laboratory goal of reporting critical results within 15 to 30 minutes after testing is complete were proposed for inpatient settings (*7*).

The aim of the study was to investigate the current performance of critical value reporting in clinical laboratories in China. Accordingly, suggestions on good quality practices of critical value reporting policy and time frames are expected to be provided.

## Materials and methods

### Survey design

The National Centre for Clinical Laboratories (NCCL) in China initiated a cross-sectional survey about critical value reporting performance. It started in March and ended in April of 2015. A questionnaire related to critical value reporting policy was sent to 1589 clinical laboratories in China online. Participants of the survey were laboratories that participate in routine chemistry external quality assessment (EQA) programs organized by NCCL. Most of the laboratories operate within Class 3A hospitals (first-class hospitals with more hospital beds and higher medical quality in China), followed by Class 3B hospitals, Class 2 and primary hospitals. Clinical laboratories from general hospitals were also included while independent commercial laboratories were excluded, as these might apply a completely different critical value reporting policy. The survey data were collected between March and April 2015.

The questionnaire consisted of two parts. The first part included the demographic data of participating laboratories and critical value reporting policies adopted. The demographic data of participating laboratories included hospital grade and number of hospital beds, laboratory accrediting information and data on laboratory information system (LIS) used. The data on critical value reporting policy included repeated testing, recording, and reporting of critical values. Using computer systems to report critical values refers to the use of LIS and hospital information systems (HIS) to report critical values. In order to compare the performance of laboratories of different grade and laboratories pertaining to hospitals with different bed numbers, laboratories were divided into several subgroups. Subgroups included: a) laboratories from Class 3A hospital, Class 3B hospital, and, Class 2 or primary hospital; b) laboratories pertaining to hospitals of 0 to 1000, 1001 to 2000, and, more than 2001 beds; and c) ISO 15189 or CAP accredited laboratories and the non-accredited ones.

The second part of the questionnaire aimed to collect information on timeliness of critical value reporting for inpatient, outpatient and emergency (STAT) patient in each laboratory during one month. This included the number failures to notify critical values, number of critical values notified after a consensually agreed time (from result validation to result communication to the clinician), number of failures to obtain receipt acknowledgement from clinicians after critical value notification, median and 90th percentiles (P_90_) of time from result validation to result communication and total number of critical values communicated over the same period.

Data collected in the second part of questionnaire was used to calculate quality indicators (QIs) based on formulas presented in Table 1. Five QIs were included: a) critical value unreported rate; b) critical value unreported timely rate; c) critical value clinical unacknowledged rate; d) median of critical value reporting time; and e) P_90_ of critical value reporting time (from result validation to result communication to the clinician). The reasons for unreported critical values and the target critical value reporting time were also collected. Invitation, primary coverage and submission path of this investigation were notified via short messages and paper letters to the laboratory leaders or quality managers. They were also asked to read instructions about data collection and questionnaire fulfilling online before uploading. Laboratories were advised to collect related data with the use of LIS and HIS. Those that did not have LIS or HIS collected related data by accessing to related records manually. Laboratory leaders or quality managers were asked to upload related data via online EQA platform for critical value reporting investigation developed by the NCCL within 2 month after receiving it voluntarily (*6*, *8*, *9*).

**Table 1:** Quality indicators and corresponding calculation formulas included in the national survey

**Quality indicator**	**Calculation formula used to obtain the quality indicator**
Critical value unreported rate	Number of failures to notify critical values divided by total number of critical values communicated over the same period.
Critical value unreported timely rate	Number of critical values notified after a consensually agreed time (from result validation to result communication to the clinician) divided by total number of critical values communicated over the same period.
Critical value clinical unacknowledged rate	Number of failure to obtain receipt acknowledgement from clinicians after critical value notification divided by total number of critical value notified.
Median of critical value reporting time	Median of time from result validation to result communication to the clinician.
P_90_ of critical value reporting time	P_90_ of time from result validation to result communication to the clinician.
The indicators were calculated for each laboratory from corresponding data provided by each laboratory. P_90_ - 90th percentile.	

### Statistical analysis

EXCEL (Microsoft, Redmond, WA) (2007 version) and SPSS 13.0 (SPSS Inc., Chicago, USA) were used to analyse the data collected. Normality was tested for all variables by Kolmogorov-Smirnov test and the distribution results were expressed as the 5th (P_5_), 25th (P_25_), 50th (P_50_), 75th (P_75_) and the 95th percentiles (P_95_).

Error rates values were calculated for critical value unreported rate; critical value unreported timely rate; and critical value clinical unacknowledged rate. Calculation formulas of error rates are shown in Table 1. Results on the error rates were expressed as percentages. In order to evaluate performance on critical value reporting, six sigma was calculated by use of an on line sigma calculator (*10*). Six-sigma represents a word-class quality with 99.9997% of the products free of defects, while 3-sigma means the performance of the procedure is 93.3193% perfect and sigma values < 3 sigma were not acceptable (*11*).

To test the differences between different subgroups of patients and hospitals, Kruskal-Wallis and Mann-Whitney U rank sum tests were used. Kruskal-Wallis rank sum test was used to obtain differences among 3 subgroups of data, whereas Mann-Whitney U rank sum test was used to obtain the difference between two subgroups of data. P ≤ 0.05 was chosen as the threshold of significance. If significant differences (P ≤ 0.05) were detected by Kruskal-Wallis rank sum test, Mann-Whitney U rank sum test was employed to find out the data of which two subgroups were significant different, under this circumstances P ≤ 0.017 was chosen as the threshold of significance.

## Results

A total of 973 laboratories submitted fulfilled questionnaires online. The response rate was 61.2% (973/1589). The demographic data of participating laboratories and their critical value reporting policies are presented in Table 2.

**Table 2:** Questions and answers of national survey on critical value reporting in China

**Questions and possible answers**	**Number of laboratories (%)**
**1.Which grade does your hospital belong to?**	
A. Class 3A hospital	592 (60.8)
B. Class 3B hospital	179 (18.4)
C. Class 2 or primary hospital	202 (20.8)
**2. How many beds does your hospital occupy?**	
A. 0 - 1000	551 (56.6)
B. 1001 - 2000	316 (32.5)
C. More than 2001	106 (10.9)
**3. Is your laboratory accredited according to ISO 15189 or CAP?**	
A. Yes	102 (10.5)
B. No	871 (89.5)
**4. Is a laboratory information system applied in your laboratory?**	
A. Yes	953 (98.0)
B. No	20 (2.1)
**5. Has a critical value reporting policy been established in your laboratory?**	
A. Yes	961 (98.8)
B. No	12 (1.2)
**6. Is repeated testing performed before calling a critical value in your laboratory?**	
A. Yes	922 (94.8)
B. No	51 (5.2)
**7. If the repeated result is not significantly different, which value is reported?**	
A. First result	633 (68.7)
B. Second result	147 (15.9)
C. Average of results	107 (11.6)
D. Others	35 (3.8)
**8. If the repeated result is not significantly different but no longer classified as a critical value, is the critical value still reported?**	
A. Yes	374 (40.6)
B. No	548 (59.4)
**9. If the repeated result is significantly different but no longer classified as a critical value, is the critical value still reported to the clinician?**	
A. Yes	48 (5.2)
B. No	91 (9.9)
C. It depends on the third repeated result	783 (84.9)
**10. Is a critical value reporting document mandatory in your laboratory?**	
A. Yes	958 (98.5)
B. No	15 (1.5)
**11. How do you record critical value reporting in your laboratory?**	
A. Computer system	122 (12.7)
B. Paper recording	293 (30.6)
C. Both A and B	543 (56.7)
**Questions and possible answers**	**Number of laboratories (%)**
12. Which items are included in critical value reporting documents in your laboratory*?	
A. Patient’s name	956 (99.8)
B. Patient’s ID	735 (76.7)
C. Patient’s ward and bed number	778 (81.2)
D. Examination and result that was reported	942 (98.3)
E. Person who received the report of the result	913 (95.3)
F. Person in the laboratory who reported the result	860 (89.8)
G. Time the result was verified and available for call–back	518 (54.1)
H. Time the result was reported	862 (90.0)
I. Verification that the verbal report was recorded accurately by the recipient (read-back)	518 (54.1)
13. How do you report critical values*?	
A. Phone calls	932 (95.8)
B. SMS	92 (9.5)
C. Computer system^†^	525 (54.0)
**14. Were rapid changes in laboratory results included in critical list in your laboratory?**	
A. Yes	239 (24.6)
B. No	734 (75.4)
ISO - international organization for standardization. CAP - College of American Pathologists. ID – identification. SMS - Short Message Service (critical values reported through sending short text messages to responsible caregivers). *More than one answer to the question could be provided. ^†^ Refers to the use of laboratory information systems and hospital information systems to report critical values.	

### Quality indicators

Results on the error rates, sigma values (σ), and reporting time for the QIs and target critical value reporting time included in the survey are shown in Table 3. The critical value unreported rate, unreported timely rate, and reporting clinical unacknowledged rate of more than half of laboratories were 0 (6σ). However, there still were nearly 5.0% of laboratories showing unacceptable performance with σ values below 3 (Table 3). More than 75.0% of participants could report half of critical values to clinicians within 20 minutes and 90% of critical values to clinicians within 25 minutes (Table 3).

**Table 3:** Results on the error rates, sigma values, reporting time for quality indicators along with target critical value reporting time obtained among different category of patients in the national survey of critical value reporting

**Patient category**	**Number of laboratories (N)**	Distribution of results*					P*	**P^†^**
**P_5_**	**P_25_**	**P_50_**	**P_75_**	**P_95_**
**Quality indicators included in the survey**								
Critical value unreported rate								
Inpatient	918	0.0 (3.0)	0.0 (6.0)	0.0 (6.0)	0.0 (6.0)	6.7 (6.0)	0.002	0.002^‡^
Outpatient	635	0.0 (3.0)	0.0 (6.0)	0.0 (6.0)	0.0 (6.0)	6.3 (6.0)		0.284^§^
Stat patient	645	0.0 (2.9)	0.0 (6.0)	0.0 (6.0)	0.0 (6.0)	7.6 (6.0)		0.043^||^
Critical value unreported timely rate								
Inpatient	792	0.0 (2.3)	0.0 (6.0)	0.0 (6.0)	3.0 (6.0)	22.7 (6.0)	< 0.001	< 0.001^‡^
Outpatient	543	0.0 (2.3)	0.0 (6.0)	0.0 (6.0)	0.0 (6.0)	20.2 (6.0)		0.414^§^
Stat patient	572	0.0 (2.4)	0.0 (3.4)	0.0 (6.0)	0.0 (6.0)	17.4 (6.0)		< 0.001^||^
Critical value reporting clinical unacknowledged rate								
Inpatient	877	0.0 (2.2)	0.0 (6.0)	0.0 (6.0)	0.0 (6.0)	25.8 (6.0)	< 0.001	< 0.001^‡^
Outpatient	603	0.0 (2.3)	0.0 (6.0)	0.0 (6.0)	0.0 (6.0)	20.5 (6.0)		0.442^§^
Stat patient	604	0.0 (2.9)	0.0 (6.0)	0.0 (6.0)	0.0 (6.0)	18.5 (6.0)		< 0.001^||^
Median of critical value reporting time in minutes								
Inpatient	686	1.0	5.0	10.0	20.0	60.0	0.004	0.011^‡^
Outpatient	641	0.0	4.0	8.0	18.0	60.0		0.656^§^
Stat patient	627	0.0	4.0	8.0	15.0	54.2		0.002^||^
P_90_ of critical value reporting time in minutes								
Inpatient	678	1.0	7.0	12.0	25.0	85.1	0.004	0.008^‡^
Outpatient	610	0.0	5.0	10.0	24.0	81.8		0.804^§^
Stat patient	623	0.0	5.0	10.0	22.0	61.8		0.003^||^
**Target critical value reporting time**								
Inpatient	901	5.0	10.0	15.0	30.0	90.0	0.695	-
Outpatient	641	5.0	10.0	15.0	30.0	90.0		
Stat patient	643	5.0	10.0	15.0	30.0	60.0		
Error rates are presented as percentages with accompanying sigma values in parenthesis. Error rates were calculated according to formulas in Table 1, whereas sigma values were calculated using calculations provided in Reference 10. P_5_ - 5th percentile. P_25_ - 25th percentile. P_50_ - 50th percentile. P_75_ - 75th percentile. P_90_ - 90th percentile. P_95_ - 95th percentile. *Kruskal-Wallis rank sum test was used to test the differences between inpatients, outpatients and stat patients with a threshold of significance of P ≤ 0.05. ^†^Mann-Whitney U rank sum test was used to test the differences between two groups with a threshold of significance of P ≤ 0.017. ^‡^Differences between inpatient and outpatient were tested. ^§^Differences between outpatient and stat patient were tested. ^||^Differences between stat patient and inpatient were tested.								

### Comparison of different groups

Significant differences were detected between laboratories occupying different bed number for critical value unreported rate of stat patient and critical value unreported timely rate, as shown in Table 4. There were no significant differences between QIs reported by laboratories from different hospital grade, and ISO 15189 or CAP accredited laboratories and the non-accrediting ones (P > 0.05).

**Table 4:** Critical value unreported rate and critical value unreported timely rates among different category of patients admitted to laboratories in different hospitals participating in the national survey of critical value reporting

**Patient category of patients**	**Number of hospital beds (N)**	**Number of laboratories (N)**	**Distribution in percentiles**						P*		**P^†^**
**P_5_**	**P_25_**	**P_50_**	**P_75_**		**P_95_**
**Critical value unreported rate**											
Stat patient	0-1000	354	0.0	0.0	0.0		0.0	2.8	0.001	< 0.001^‡^	
	1001-2000	222	0.0	0.0	0.0		0.0	18.0		0.304^§^	
	> 2001	69	0.0	0.0	0.0		0.0	12.0		0.200^||^	
**Critical value unreported timely rate**											
Inpatient	0-1000	450	0.0	0.0	0.0		2.3	19.6	< 0.001	0.003^‡^	
	1001-2000	256	0.0	0.0	0.0		5.0	23.0		0.173^§^	
	> 2001	86	0.0	0.0	0.0		0.0 0.0 2.0	30.4		< 0.001^||^	
Outpatient	0-1000	299	0.0	0.0	0.0			14.2	0.001	0.001^‡^	
	1001-2000	187	0.0	0.0	0.0			28.7		0.577^§^	
	> 2001	57	0.0	0.0	0.0		4.7	34.3		0.004^||^	
Stat patient	0-1000	308	0.0	0.0	0.0		5.9	15.6	0.001	0.002^‡^	
	1001-2000	205	0.0	0.0	0.0		2.4	20.7		0.377^§^	
	> 2001	59	0.0	0.0	0.0		6.5	30.7		0.003^||^	
Only quality indicators with Kruskal-Wallis rank sum test P value ≤ 0.05 are presented. The error rates are presented as percentages. P_5_ - 5th percentile. P_25_ - 25th percentile. P_50_ - 50th percentile. P_75_ - 75th percentile. P_90_ - 90th percentile. P_95_ - 95th percentile. *Kruskal-Wallis rank sum test was used to test the differences among hospitals occupying 0 -1000, 1001-2000 and more than 2001 beds; P ≤ 0.05 was chosen as the threshold of significance. ^†^Mann-Whitney U rank sum test was used to test the differences between two groups, P ≤ 0.017 was chosen as the threshold of significance. ^‡^Differences between hospitals with 0-1000 and 1001-2000 beds were tested. ^§^Differences between hospitals with 1001-2000 and > 2001 beds were tested. ^||^Differences between hospitals with > 2001 beds and 0-1000 beds were tested.											

### Reasons for unreported critical value

Reasons submitted by participants for unreported critical value among outpatient, inpatient and stat patient are shown in Table 5.

**Table 5:** Reasons for unreported critical value submitted by participants in the national survey of critical value reporting

**Reason for unreported critical value**	**Occurrence (N)**		
**Inpatient**	**Outpatient**	**Stat patient**
Reporting omission caused by laboratory staff	111	32	49
Communications equipment failure to connect	104	51	37
Uncompleted application form without contact information of clinician	82	66	44
Uncompleted application form without contact information of outpatient	0	11	0

## Discussion

Timeliness and accuracy of critical value reporting are important aspects that should be ensured in clinical laboratories. The National Patients Safety Goals in China in 2007, Hospital Management Evaluation Guideline, as well as the Evaluation Standards for General Hospitals all required the timeliness reporting of critical values (*4*). Majority of participants in this survey were from Class 3 hospitals in China. In order to fulfil these national requirements, great attention has been drawn to critical values reporting policies. Based on this situation, the critical value unreported rate, unreported timely rate, and clinical unacknowledged rate of more than half of participants were satisfying with zero error rates. More than 75.0% of participants could report half of critical values to clinicians within 20 minutes and could report 90% of critical values to clinicians within 25 minutes.

A CAP Q-Probes study of 121 institutions showed that laboratories needed a median of 5 minutes for staff to notify critical values from result available to reporting complete, calling critical results within 15 to 30 minutes after completed testing. This period was proposed as target timeframe for inpatient setting in the same survey (*7*). Recommendations of communicating critical test results proposed by Massachusetts Hospitals also give suggestions on critical value reporting time frames. They believe notification time parameters for communicating critical test results should be designed according to urgency, *e.g.* within 1 hour, within the shift (target 6-8 hours), within 3 days (*12*). As more than 75.0% of participants in our survey could report 90.0% of critical values to clinicians within 25 minutes, 25 minutes (from result validation to result communication to the clinician) was recommended as a target time to encourage improvements in the rest of the laboratories investigated. However, authors believe laboratories should set their target critical value reporting timeframes with clinicians in their institutions to fulfil clinical need, 25 minutes was only a reference.

A CAP Q-Track study assessed the frequency of unreported critical values in 180 institutions with different size and scope. The median rates of communication failure (critical values without documented report to a responsible caregiver) were higher for outpatients than inpatients (*13*). Other studies also indicated that the biggest obstacle to successful critical value reporting was outpatient physician not returning calls or pagers (*14*). On the contrary, data in this survey showed that critical value unreported rate of inpatients were significantly higher than that of outpatients; similar results were obtained for critical value unreported timely rate and clinical unacknowledged rate. Furthermore, the critical value reporting time of inpatients was significant longer than for outpatients. Low critical value incidence with less reporting workload for outpatient may partly explain this result. Significant differences for critical value reporting performance were detected between laboratories pertaining to hospitals with different bed numbers. Laboratories in hospitals with less bed numbers seemed to have better performances than laboratories in hospitals with more beds. Heavy workload in large hospital may also be related. On one hand, laboratory staff in laboratories with heavy workload is always busy with testing patients’ specimens and this may delay the reporting of critical values they detected. On the other hand, it may be a more difficult task to contact busy responsible caregivers in large hospitals than in small ones. Call centres with dedicated staff to report critical values or electronic communications systems were recommended in laboratories with larger workload (*5*).

“Reporting omission caused by laboratory staff”, “communications equipment failure to connect”, and “uncompleted application form without contact information of clinician” were the three major reasons for unreported critical values. Training activities are needed for laboratory staff to realize the importance of timely and accurate reporting of critical values in case of staff omissions. A backup plan for LIS shutdown and complete application information in HIS are also needed to avoid unreported critical values caused by “communications equipment failure to connect” and “uncompleted application form without contact information of clinician”.

The majority of laboratories repeat critical value tests before reporting to responsible caregivers. Munoz evaluated the efficacy of repeating critical values in the United States, and found that 68.2% of laboratories always repeat critical results before reporting (*15*). In a CAP Q-Probes study, 48 out of 86 laboratories always repeated chemistry critical values before reporting (*16*). In this survey, most (94.8%) of participants implemented this practice in their laboratory as well. However, this practice was generally established to assure accuracy of critical results when manual methods were dominant while laboratory automation and standardization were not prevalent (*17*). Laboratory may repeat examinations of such critical values before verification to avoid analysis errors. However nowadays several research groups have determined that automated critical values are accurate using modern equipment and methods, and that repeated examinations caused significant delays in reporting (*18*, *19*). Critical values obtained with modern instrumentation are likely to be valid when they fall within the reportable range of the analyzer and do not fail delta check rules (*5*). This practice may cause reporting delays without significant benefit and it should be carefully evaluated for its usefulness before being implemented.

Although electronic patient records, LIS and HIS have been introduced in hospitals widely, the most common reporting system is still telephone communication with the recipient expected to verify accuracy by documenting and reading back the report. It is worth noting that more than half of participants have employed computer system (LIS and HIS) to report critical value. Evidence showed that digital, non-telephonic systems seemed to be timelier and more accurate compared to telephone communication (*20*-*22*). However, the receipt of critical value by responsible caregivers should be assured and documented if digital systems are used to replace telephone.

Only 24.6% of participants included rapid changing results in their critical lists. As these results may also indicate life-threatening situations, laboratories should pay more attention to avoid neglecting these situations when only critical limits are employed. Therefore, laboratories were advised to set multiple rules in critical value list and import them in LIS to help identifying rapid change of test results that may indicate life-threatening situations.

Nowadays, there were many common terms used in different literature including “critical result”, “critical value”, “panic value”, “crisis value” and “alarm value” (*14*, *23*). The CLSI document GP47 uses the term “critical-risk result” rather than “critical value” in order to emphasize the focus on the risk level to patients, rather than the degree of the result abnormality (*5*, *24*). As only quantitative items were included in this survey meanwhile “critical value” was widely used in China, “critical value” was employed here.

Although critical value reporting policies has been investigated before (*4*), there was not any investigation on the timeliness of critical value reporting in different laboratories ever launched in China. This investigation can provide reference for the laboratory setting critical value reporting timeframe and working out good quality practices. However, there were several limitations. Firstly, as recognition time can barely be recorded in LIS, critical value reporting time in this survey refers to time from result validation to result communication to clinician which did not contain time from recognition to validation. Monitoring time from critical value recognition to validation was encouraged in laboratory if possible. Secondly, the major participants were from tertiary hospitals in China, so the results represent laboratories with more critical values and heavier workload in China. Additionally, although it was a voluntary, free of charge and non-punitive investigation and laboratories were encouraged to submit truthful data, the truthfulness of data submitted by participants in this survey may still be questioned. Besides, reasons submitted by participants for unreported critical value depending on number of laboratories provided that answer. Finally, this is a short-time survey and the data was collected only for one month, thus the robustness of error rates and sigma values we calculated was uncertain. Laboratories were advised to strengthen the construction of information systems to monitor critical value reporting process, set suitable timeframe and identify reasons for critical value reporting delay through lengthways internal monitoring and transverse external comparison.

In conclusion, the majority of laboratories can report critical values to responsible clinical staff within 25 minutes. Thus, in order to improve reporting of critical results of biochemistry analyses in most laboratories in China this value could be recommended as suitable reporting timeframe. However, along with timeframe setup, careful monitoring of the complete reporting process and improvement of the information systems could significantly contribute to timeliness in critical value reporting.
